# *TitrationAnalysis*: a tool for high throughput binding kinetics data analysis for multiple label-free platforms

**DOI:** 10.12688/gatesopenres.14743.2

**Published:** 2024-06-28

**Authors:** Kan Li, Richard H.C. Huntwork, Gillian Q. Horn, S. Munir Alam, Georgia D. Tomaras, S. Moses Dennison

**Affiliations:** 1 Center for Human Systems Immunology, Duke University, Durham, North Carolina, 27701, USA; 2 Department of Surgery, Duke University, Durham, North Carolina, 27710, USA; 3 Department of Pathology, Duke University, Durham, North Carolina, 27710, USA; 4 Duke Human Vaccine Institute, Duke University, Durham, North Carolina, 27710, USA; 5 Department of Molecular Genetics and Microbiology, Duke University, Durham, North Carolina, 27710, USA; 6 Department of Integrative Immunobiology, Duke University, Durham, North Carolina, 27710, USA

**Keywords:** Surface Plasmon Resonance, Biolayer Interferometry, antibody binding, high-throughput kinetics analysis, non-linear curve fitting

## Abstract

Label-free techniques including Surface Plasmon Resonance (SPR) and Biolayer Interferometry (BLI) are biophysical tools widely used to collect binding kinetics data of bimolecular interactions. To efficiently analyze SPR and BLI binding kinetics data, we have built a new high throughput analysis tool named the *TitrationAnalysis*. It can be used as a package in the Mathematica scripting environment and ultilize the non-linear curve-fitting module of Mathematica for its core function. This tool can fit the binding time course data and estimate association and dissociation rate constants ( *k _a_* and *k _d_* respectively) for determining apparent dissociation constant ( *K _D_*) values. The high throughput fitting process is automatic, requires minimal knowledge on Mathematica scripting and can be applied to data from multiple label-free platforms. We demonstrate that the *TitrationAnalysis* is optimal to analyze antibody-antigen binding data acquired on Biacore T200 (SPR), Carterra LSA (SPR imaging) and ForteBio Octet Red384 (BLI) platforms. The *k _a_*, *k _d_* and *K _D_* values derived using *TitrationAnalysis* very closely matched the results from the commercial analysis software provided specifically for these instruments. Additionally, the *TitrationAnalysis* tool generates user-directed customizable results output that can be readily used in downstream Data Quality Control associated with Good Clinical Laboratory Practice operations. With the versatility in source of data input source and options of analysis result output, the *TitrationAnalysis* high throughput analysis tool offers investigators a powerful alternative in biomolecular interaction characterization.

## Introduction

Label-free techniques including Surface Plasmon Resonance (SPR), Surface Plasmon Resonance Imaging (SPRi), and Biolayer Interferometry (BLI) for monitoring biomolecular interactions ( *e.g.*, antigen-antibody ^1^ or lipid-protein ^2^) in real time have become ubiquitous for kinetics characterization ^3– 6^. Currently high-throughput SPR, SPRi and BLI platforms have enabled the simultaneous detection of up to 384 biomolecular interactions leading to a wealth of kinetics data ^4^.

Briefly, SPR spectroscopy operates on the principle of total internal reflection of linear polarized light passing through the interface of two mediums with different optical densities ( *e.g.*, a thin metal film and liquid) ^2, 3, 7– 10^. The incident light couples with freely oscillating electrons within the thin metal film at a specific angle (known as the resonance angle) generating a non-radiative evanescent electromagnetic wave parallel to the surface of the thin metal film, leading to plasmon excitation ^2, 3, 7– 10^. The resonance angle is sensitive to the refractive index of the less optically-dense medium (commonly a liquid buffer) at the thin metal film-liquid interface ^2, 3, 7– 10^. The refractive index at the interface is affected by the liquid buffer properties including pH, salt concentration, and viscosity as well as mass changes on the surface ^2, 3, 7– 10^. Thus, binding events between molecules in liquid (analytes) and molecules immobilized on the surface of thin metal films (ligands) can be directly observed through continuously monitoring shifts in the resonance angle ^1– 3, 7– 10^, with the output signals recorded in resonance unit (RU). The background signal contributed by the buffer and non-specific interactions between the analytes and the sensor surface without ligands can be eliminated through reference subtraction using parallel data collected on a reference surface ^7^. Similarly, BLI monitors the change in wavelength shift in the interference pattern of white light reflected off a biolayer (a functionalized layer of immobilized ligands) and an internal reference layer located at the tip of a fiber optic sensor ^11, 12^. The change in wavelength shift occurs due to changes in the thickness of the biolayer resulting from the adsorption or desorption of analytes ^12^.

Recently, there are also newly emerged label-free techniques that have shown to provide unique advantages. Grating-coupled interferometry (GCI) and focal molography are worthy examples, both of which involve the use of a tantalum pentoxide (Ta _2_O _5_) thin-film optical waveguide ^13, 14^. GCI uses interference-based waveguide sensors ^13, 15^: the reference arm of the interferometer is combined with the measurement arm to eliminate phase noise and fluctuations. GCI exhibited high sensitivity and was shown to be particularly useful for low molecular size analyte under 1000 Da ^13, 16^. In focal molography, ligands are precisely assembled in to a specific spatial pattern (molecular hologram) to diffract light coherently, leading to the detection of signal change when bound by a specific target ^14, 17^. The noncoherent surroundings do not create coherent diffraction signal, therefore greatly reduce the detection of nonspecific binding ^14^. This enables the measurements of molecular interaction directly in biological relevant solutions, such as serum or plasma samples, as well as the detection of protein in living cell cultures ^14^.

The binding responses on label-free kinetics platforms are typically continuously monitored over time resulting in a binding time course (response unit vs. time or shift in wavelength in nanometer vs. time) commonly known as a sensorgram. Typically, the reference subtracted binding time courses are fit to a Langmuir 1:1 kinetics model ^8^ for the global estimation of kinetics parameters including association and dissociation rate constants ( *k _a_* and *k _d_* respectively) for the determination of the apparent dissociation equilibrium constant ( *K _D_*) values. More complicated kinetics models can be implemented for more complex bindings. These models include mass-transport limited ^18, 19^, bivalent analyte ^20^, heterogeneous ligand ^20^, heterogeneous analyte ^21^, and two-state ^22^ models.

The estimated *k _a_*, *k _d_*, and *K _D_* values along with other biophysical data can provide key insights into features of biomolecular interactions such as epitope recognition of antibodies and ligand binding to receptors ^23^. For example, antibody affinity/avidity and epitope specificity can quickly be assessed through kinetics titrations of antigens as analytes on SPR, SPRi, and BLI platforms ^24, 25^. This is a crucial step for the identification and development of therapeutic antibody candidates ^26, 27^.

Currently, besides commercial analysis software that are typically accompanying the platform instruments, there are some packages and software for third party use. These include commercial software such as Scrubber and TraceDrawer as well as freely available software Anabel ^5^. However, processing and analyzing high volume of kinetics data can be non-uniform, cumbersome, and inefficient especially for a large panel of biomolecules with diverse kinetics behaviors ^28, 29^. Furthermore, it can be challenging for laboratories operating under Good Clinical Laboratory Practice (GCLP) guidelines ^30^ that report binding kinetics data with stringent Quality Control (QC) criteria to quickly collate high quality binding kinetics data analysis reports in a custom format for record keeping and filing in a streamlined fashion. Given the broad array of SPR, SPRi, BLI and other label-free kinetics platforms available, it is advantageous for investigators to have a binding kinetics analysis package that has cross-platform compatibility ^5^, ability to batch process tens to hundreds of binding time courses, and options for versatile and customizable user-guided data processing and reporting.

For these reasons, we developed *TitrationAnalysis*, a Mathematica package for automated and high throughput kinetics analysis of binding time courses. *TitrationAnalysis* tool, which currently focuses binding kinetics analysis for Biacore T200 (SPR), Carterra LSA (SPRi), and Fortebio Octet Red384 (BLI) platforms, is available for free and incorporates the “best of *”* kinetics analysis features found in a number of commercial kinetics analysis platforms and requires minimal knowledge for Mathematica scripting. Mathematica is a software with robust computation abilities and was chosen here as the scripting environment for the *TitrationAnalysis* tool due to its broad accessibility, particularly to academic researchers. The tool automatically fits each included sensorgram after the user provides exported binding time courses and user-defined fitting parameters. The reports the *TitrationAnalysis* tool generates incorporate user-directed options and include information that can be readily used for downstream data quality control and reporting. The quality of *TitrationAnalysis* derived *k _a_*, *k _d_*, and *K _D_* values can be assessed based on fitted residuals and standard errors. In this work we have demonstrated the utility of *TitrationAnalysis* through the kinetics analysis of the interactions between a HIV-1 neutralizing monoclonal antibody (CH31) ^31^ and a HIV-1 envelope glycoprotein (AE.A244 gp120) ^32^ collected across the Biacore T200 (SPR), Carterra LSA (SPRi), and Fortebio Octet Red384 (BLI) platforms, where the *TitrationAnalysis* derived *k _a_*, *k _d_*, and *K _D_* values were in close agreement with the native commercial software.

## Analytical methods

### Mathematical model for tool development

The *TitrationAnalysis* tool was built upon Mathematica v12.0 and can be easily adapted for Mathematica v13.0. The package as well as example input and output files can be accessed at https://github.com/DukeCHSI/TitrationAnalysis and at https://zenodo.org/record/7998652 ^33^.

The *TitrationAnalysis* tool uses Equation 1 and Equation 2 shown below to fit the sensorgrams to a 1:1 Langmuir binding model. The tool provides the option to handle non-regenerative titrations (alternatively known as single cycle kinetics) that do not include a regeneration step between cycles.

The linear equation for fitting association data and dissociation data are:

$$\text{Association}:R_{t}=R_{shift}^{i}+R_{max}^{i}\times \frac{k_{a}\times C_{i}}{k_{a}\times C_{i}+k_{d}}\times (1-e^{-(k_{a}\times C_{i}+k_{d})\times (t-t_{0}^{i})})\;(1)$$

$$\text{Dissociation}:R_{t}=R_{drift}^{i}+(R_{max}^{i}\times \frac{k_{a}\times C_{i}}{k_{a}\times C_{i}+k_{d}}\times \left(1-e^{-(k_{a}\times C_{i}+k_{d})\times (t_{asso}-t_{0}^{i})}\right))\times e^{-k_{d}\times (t-t_{asso})}\;(2)$$

Here *R _t_* is the response at time *t*. *C _i_* is the molar concentration of analyte in cycle *i*, *R _max_* is the maximal response feasible. *k _a_* is the association rate constant, *k _d_* is the dissociation rate constant and *t _asso_* is the absolute time when association ends. In non-regenerative titration fitting, $t_{0}^{i}$ fits for the extrapolated time where the response is 0 for analyte cycle *i*; in regenerative titration fitting, $t_{0}^{i}$ becomes a fixed value *t*_0_, corresponding to the absolute time when the association starts. In local *R _max_* fitting, $R_{max}^{i}$ fits for *R _max_* value for analyte cycle *i*, and becomes a non-local *R _max_* in the case of global *R _max_* fitting. $R_{shift}^{i}$ is optional and fits bulk shift at the start of the association. This bulk shift is typically due to a mismatch between the analyte buffer and the running buffer used for collecting baseline and dissociation data, and will therefore typically disappear when association ends. This causes a signal disconnect both at the beginning and at the end of the association phase. $R_{drift}^{i}$ is optional and accounts for quick change in signal at the beginning of dissociation, due to factors such as the loss of non-specifically bound analyte. To avoid over-parameterization, $R_{drift}^{i}$ term will be dropped if $R_{shift}^{i}$ term is included. In practice, Equation 2 is modified to Equation 3, which produces identical kinetics and *R _max_* estimations and has more stable fitting performance than Equation 2. Table 1 summarizes the parameter details.

$$\text{Dissociation}:R_{t}=(R_{max}^{i}+R_{drift}^{i})\times \frac{k_{a}\times C_{i}}{k_{a}\times C_{i}+k_{d}}\times \left(1-e^{-(k_{a}\times C_{i}+k_{d})\times (t_{asso}-t_{0}^{i})}\right)\times e^{-k_{d}\times (t-t_{asso})}\;(3)$$

**Table 1.** Detailed explanations of parameters in kinetics equations used in the *TitrationAnalysis* tool.

Parameter	Definition	Fixed or Floated	Note
***C _i_***	molar concentration of analyte in cycle *i*	Fixed	Known through assay design
***t _asso_***	absolute time when association ends	Fixed	Known through assay design
***R _max_***	theoretical maximal response	Floated	Used in global *R _max_* fitting
$R_{max}^{i}$	theoretical maximal response for cycle *i*	Floated	Used in local *R _max_* fitting
***k _a_***	association rate constant	Floated	Kinetics parameter
***k _d_***	dissociation rate constant	Floated	Kinetics parameter
$R_{shift}^{i}$	accounts for bulk shift at the start of the association	Floated	Used to address bulk shift
$R_{drift}^{i}$	accounts for quick change in signal at the start of dissociation	Floated	Used to address short phase of signal change
***t _0_***	absolute time when the association starts	Fixed	Known through assay design
$t_{0}^{i}$	extrapolated time where the response is 0 for analyte cycle *i*	Float	Used in non-regenerative cycle data

### Standard error estimation

Standard errors for R _max_, *k _a_* and *k _d_* estimations are calculated from “NonlinearModelFit”, the Mathematica module used for data fitting using Equation 1 and Equation 3. The parameter optimization was done through the minimization of sum of square error. The standard error for *K _D_* estimation was calculated through error propagation using the standard errors of *k _a_* and *k _d_* through Equation 4:

$$\text{Δ}K_{D}=K_{D}\times \sqrt{{\left(\frac{\text{Δ}k_{a}}{k_{a}}\right)}^{2}+{\left(\frac{\text{Δ}k_{d}}{k_{d}}\right)}^{2}}\;(4)$$

where the symbol Δ before *k _a_*, *k _d_* and *K _D_* represents the standard error of the corresponding value.

### Implementation of the TitrationAnalysis tool

**Figure 1.**
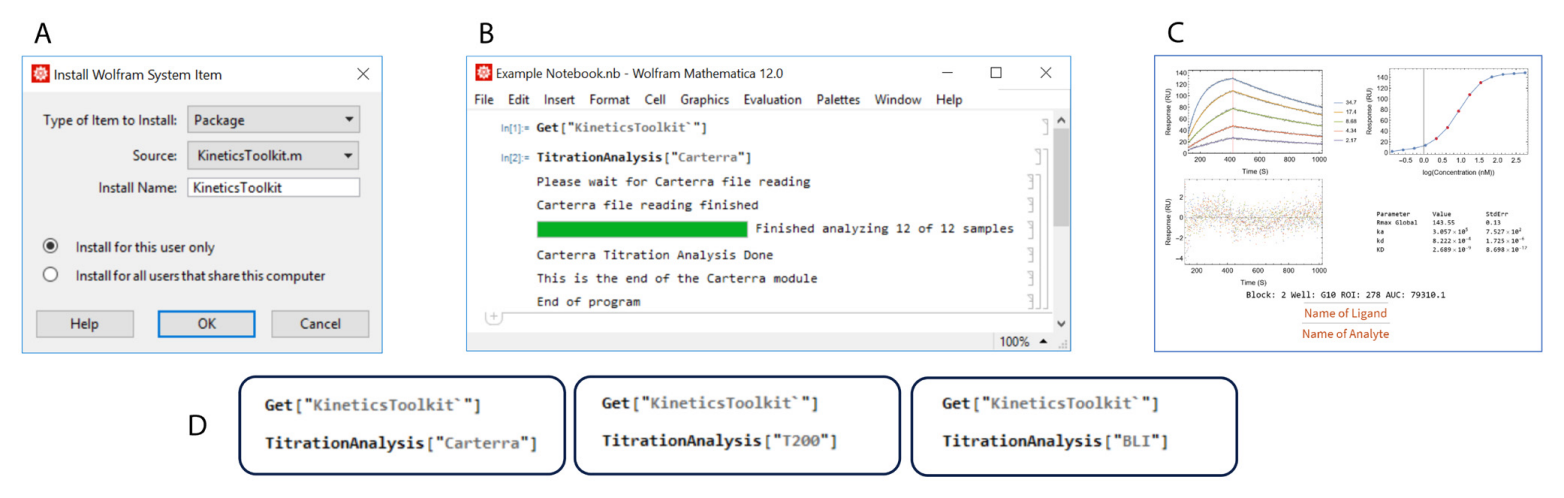
The *TitrationAnalysis* tool can batch process sensorgram fitting and automatically generate reports. This figure shows an overall schematic of the installation and execution of the *TitrationAnalysis* tool. Panel **A** shows the installation of *KineticsToolkit*, the overall package containing *TitrationAnalysis* tool. Panel **B** shows the input commands to execute the *TitrationAnalysis* tool and the output at the end of TitrationAnalysis tool execution. Panel **C** shows an example of PDF report pages automatically generated after fitting analysis. Panel **D** shows the available modules that can be called within the *TitrationAnalysis* tool to import and analyze data collected on different instruments.

Figure 1 demonstrates the installation and execution of the *TitrationAnalysis* tool. The user will need to install the package under the name “KineticsToolkit” and use the command Get["KineticsToolkit`"] to activate the package inside Mathematica. Then the *TitrationAnalysis* tool can be implemented for the appropriate platform ( Figure 1D). A series of pop-up windows will guide the user through data import and global settings before sequentially fitting sensorgrams and generating output files. More details on implementation can be found at https://github.com/DukeCHSI/TitrationAnalysis.

### Label-free platforms adaptability of *TitrationAnalysis*

*TitrationAnalysis* tool is designed to directly import a large amount of reference subtracted data exported from commercial software provided for different label-free platforms, with no or minimal reformatting. The tool has the ability to handle data from three different instruments for measuring binding kinetics data ( Figure 1): Carterra LSA for high-throughput SPR _i_, Octet Red384 for high-throughput BLI, and Biacore T200 for standard throughput SPR.

### Operation of the *TitrationAnalysis* tool

**Figure 2.**
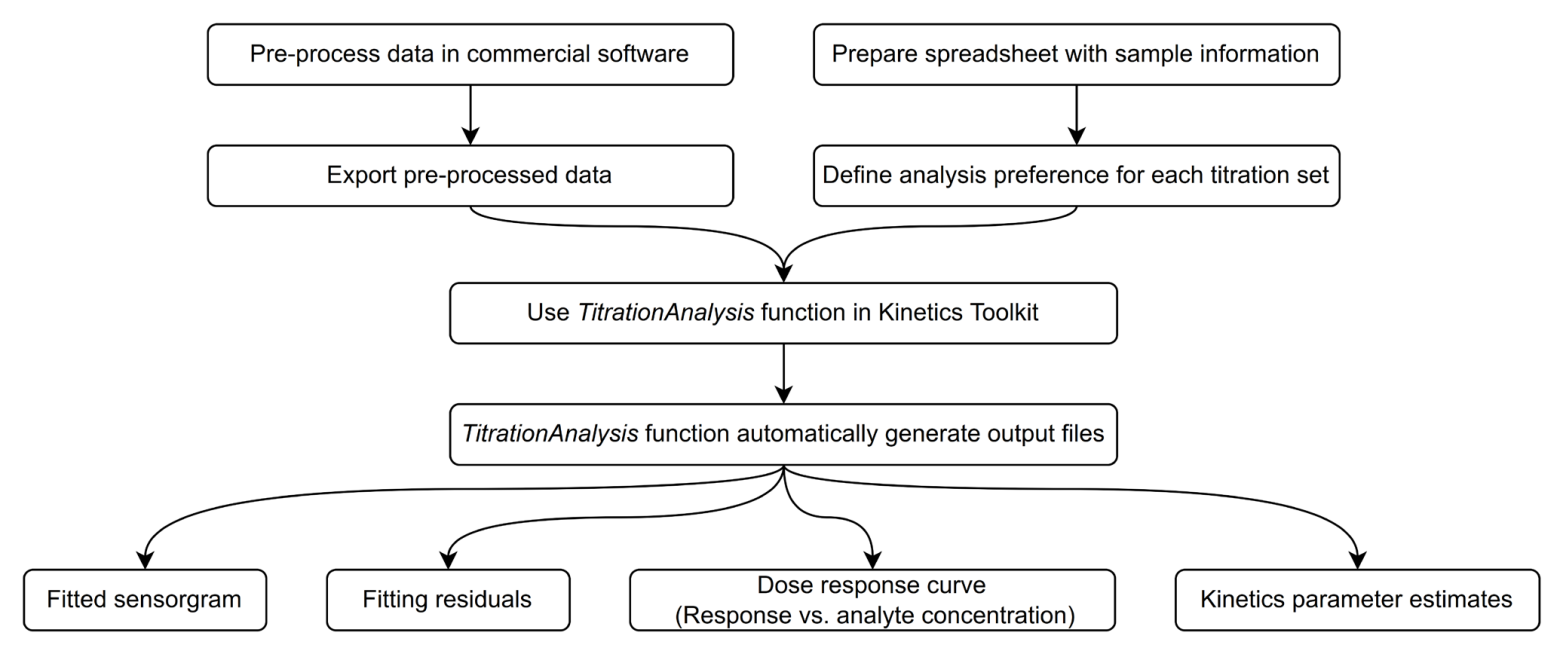
Flowchart of *TitrationAnalysis* tool data importing and results exporting.

Here we provide a general overview of how the users may typically operate the *TitrationAnalysis* tool, as shown in Figure 2. The minimal system requirements for using the *TitrationAnalysis* tool is the same as those for using the Mathematica environment overall: Windows 10 or higher, 19 GB of disk space and 4 GB of RAM ( https://support.wolfram.com/6479).

Typically, the commercial software is capable of data reference subtraction, zero analyte concentration cycle (blank cycle) subtraction and data smoothing. User is expected to do the aforementioned steps as data pre-processing and export of the pre-processed data. For data obtained on Biacore T200 and Octet Red384, the blank cycle subtraction can be done during automatic fitting using *TitrationAnalysis* tool if data for a zero analyte concentration cycle is provided and therefore is optional during pre-processing. After pre-processing, the time points and their corresponding response values can be exported as data tables in various file formats.

When calling a specific instrument module, the user will be prompted to provide the pre-processed data and a corresponding sample information spreadsheet. The content of the spreadsheet will be described in detail in section titled “Sample information and analysis preference settings”. If the content of the spreadsheet matches what is included in the pre-processed data, the tool will automatically fit each sensorgram sequentially and generate report files after all fittings are done.

Report files include a PDF report and a standalone report of parameter estimates. Each page of the PDF report will correspond to one sensorgram series associated with a given ligand surface and include the fitted sensorgram overlaid with underlying data, fitting residuals, a dose-response plot (Response at the end of association phase versus log of analyte concentration) and a summary of parameter estimates. Alongside of the PDF report, an additional report in .csv format will also lay out the details of kinetics parameter estimates, and associated standard errors of R _max_, *k _a_*, *k _d_*, and *K _D_*. The standalone .csv report can be readily used to calculate the relative standard error of each kinetics parameter estimate, the averages of the estimates among replicates and the percent coefficient of variation among replicates of the same kinetics parameter.

### Sample information and analysis preference settings

**Figure 3.**
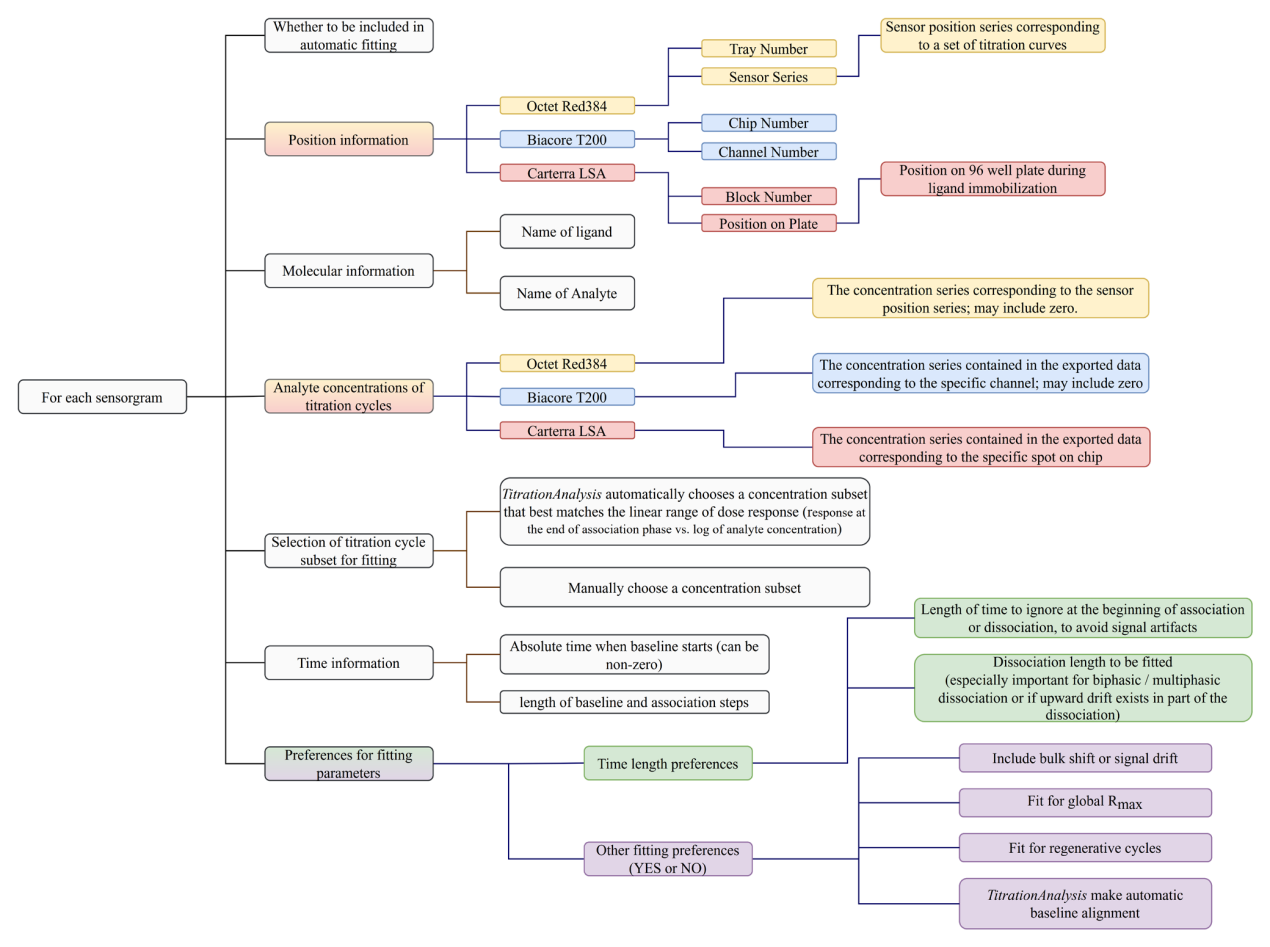
Summary of information and preferences to be provided for each sensorgram prior to automatic fitting.

A user-prepared spreadsheet with sample information and analysis preference is to be provided so that the *TitrationAnalysis* tool can correctly import and analyze as well as export fitting results. The spreadsheet can be in .csv or single tab Excel format. The information and preferences that are expected to be included in the spreadsheet are summarized in Figure 3.

Carterra LSA software is capable of simultaneously collecting titration data for up to 384 spots on a single sensor chip, and can have pre-processed data on all spots exported collectively as a single file. Biacore T200 software is capable of exporting all titration cycles from a specific channel as a single file. Octet Red384 software is capable of exporting data from each sensor as a single file. For Carterra LSA, the *TitrationAnalysis* tool requires the user to list sample information for all spots, with each spot appearing once, and choose what subset of ligands to be included in fitting. For Biacore T200 and Octet Red384, the user is only required to include relevant sensorgrams, and the same sensorgram can appear multiple times with varying analysis preferences.

### *TitrationAnalysis* internal workflow

After matching the instrumentation with the user provided information spreadsheet, for each titration series sensorgram, the *TitrationAnalysis* tool extracts data points based on the sample locations user has provided. Then the following steps will be executed to prepare data for fitting:

1. If the user chooses to have the tool make automatic baseline alignment, the appropriate baseline alignment will be made depending on whether the sensorgram was collected with regenerative or non-regenerative cycles.
2. For Biacore T200 and Octet Red384, depending on whether a zero analyte concentration cycle (blank cycle) is included in the list of cycles, optional blank cycle subtraction will be made.
3. After sorting the analyte concentrations from low to high, up to 5 analyte concentrations will be down-selected for fitting. If the user chooses to have the tool automatically select concentration range, the tool will choose the 5 consecutive analyte concentrations with the largest accumulative increase of response at end of association. This should typically closely resemble the linear range of the dose response. Otherwise the user can manually select a subset of five or fewer analyte concentrations.
4. Based on the time length information provided by the user, including the length of baseline and association, the length of dissociation to be fitted, and the length of time to be skipped over at the beginning of association and dissociation, the correct subset of data points will be selected for fitting.

**Figure 4.**
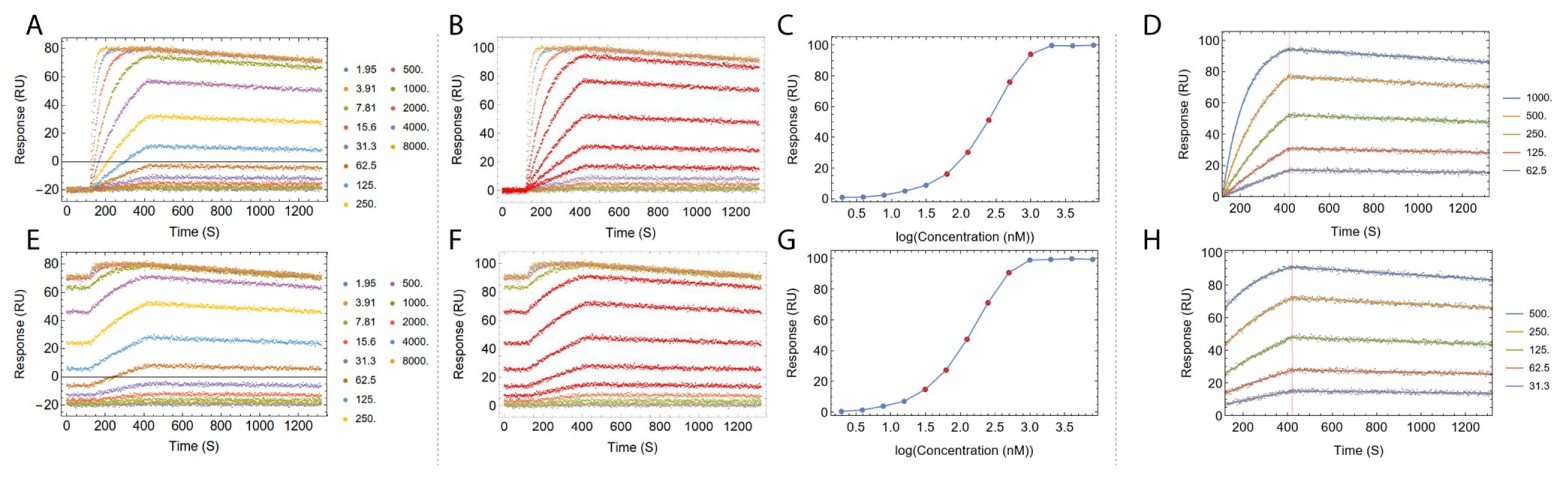
The *TitratonAnalysis* tool can do automatic baseline alignment and analyte concentration range down-selection before fitting. Fitting process of a set of simulated regenerative titration data ( **A**– **D**) and a set of simulated non-regenerative titration data ( **E**– **H**) is shown. Panels **A** and **E** show the titration data prior to baseline alignment. The color of each titration cycle and the corresponding analyte concentration in nanomolar is shown in the legend. Panels **B** and **F** show the titration after automatic baseline alignment. Panels **C** and **G** show the automatic selection of a subset of the analyte concentrations that best approximate the dose response linear range, highlighted as points in red in dose response curves, matching kinetics traces in red in panels **B** and **F**. Panels **D** and **H** show the resulting fitted sensorgram overlaid on top of underlying data points. Titration data were simulated using Equation 1 and Equation 3 with *k _a_* = 1×10 ^5^ (M ^-1^ s ^-1^), *k _d_* = 1×10 ^-4^ (s ^-1^) and R _max_ = 100 (RU).

Step 1 and 3 are depicted in Figure 4.

The Mathematica module “NonlinearModelFit” is used to call the kinetics model and conduct fitting. Depending whether the user chooses to include bulk shift, to fit for global *R _max_* or fit for regenerative cycles, the correct variation of kinetics model will be called for fitting.

## Experimental methods

### Materials

CH31 ^31, 34^ and AE.A244 gp120 ^32, 35, 36^ were produced by the Duke Human Vaccine Institute, Duke University. The transfection was done using 293 cells or CHO mammalian cells with plasmids for recombinant expression. The proteins were quality controlled for purity, including using SDS-PAGE, Western Blot and size exclusion chromatography.

### Carterra LSA data collection

Kinetics titrations were performed using HC30M sensor chips (Carterra, Part# 4279) at 25°C. Aqueous solutions were delivered onto the sensor chip using the Carterra LSA microfluidic modules, including a 96-channel print-head (96PH) and a single flow cell (SFC).

Goat anti-Human IgG Fc antibody (Millipore, Cat# AP113) was first immobilized onto the chip through amine-coupling. Briefly, the chip surface was activated using 100 mM N-Hydroxysuccinimide (NHS) and 400 mM 1-Ethyl-3-(3-dimethylaminopropyl) carbodiimide hydrochloride (EDC) (Cytiva, Cat# BR100050, mixed 1:1:1 with 0.1 M MES buffer at pH 5.5) for 600 seconds. Then anti-Human IgG Fc (in 10 mM Sodium Acetate at pH 4.5) was immobilized onto the activated surface for 900 seconds at 50 µg/ml, followed by an injection of 1 M Ethanolamine-HCl at pH 8.5 for 600 seconds to quench unreactive esters. The chip was then exposed to two 30 seconds injections of 10 mM Glycine at pH 2.0. The anti-Human IgG Fc immobilization steps were done using SFC and 10 mM MES buffer at pH 5.5 with 0.01% Tween-20 as running buffer. CH31 was then captured by the anti-Human IgG Fc at 10 µg/ml for 600 seconds using the 96PH, with 1X HBSTE buffer (10 mM HEPES pH 7.4, 150 mM NaCl, 3 mM EDTA and 0.01% Tween-20) as running buffer and antibody diluent.

A two-fold dilution series of the antigen was prepared, with the top concentration for AE.A244 gp120 being 1µM. The antigen was then injected onto the chip surface from the lowest to the highest concentration sequentially without regeneration using SFC, preceded by 8 cycles of buffer injection for signal stabilization. For each concentration, the time-length for the data collection of baseline, association and dissociation was respectively 120 seconds, 300 seconds and 750 seconds. 1X HBSTE was used as titration running buffer and sample diluent.

The titration data collected were first pre-processed in the Kinetics (Carterra) software, including reference subtraction using empty spots on the sensor chip, blank subtraction and data smoothing. The data were analyzed within Kinetics software as well as exported and analyzed using the *TitrationAnalysis* tool.

### Biacore T200 data collection

Kinetics titrations were performed using a CM5 sensor chip (Cytiva, Cat# BR100530) at 25°C. The activation of the carboxymethylated-dextran gold surface was achieved by injecting 200/50 mM EDC/NHS (Cytiva, Cat# BR100050) solution in ultrapure water pH 7.0 injected at 5 µL/min for 400 seconds. Following the activation step, a 50 µg/mL solution of anti-human IgG Fc (Millipore, Cat# AP113) in sodium acetate (NaOAc) pH 5.0 (Cytiva) was injected over the activated surface at 5 µL/min. Anti-human IgG Fc was injected in the sample channel for one injection of 120 seconds to reach ~7700 RU, and was injected in the reference channel for three injections of 200 seconds total to reach ~ 6900 RU. After covalent modification of the sensor surface, a quenching solution of ethanolamine pH 8.5 (Cytiva) was injected over the surface for 600 seconds to cap any residual active NHS esters.

PBS 1X pH 7.4 was used for the running buffer during titration. During the kinetics assay, one flow cell channel with only anti-human IgG Fc served as a reference channel to monitor and subtract binding responses due to non-specific interactions. 190–380 RU of CH31 at 5 µg/mL was captured onto the chip for each cycle at 5 µL/min for 60 seconds. The optimized capture of CH31 was followed by baseline monitoring for 60 seconds and the injection of AE.A244 gp120 for 180 seconds. Then a dissociation step was performed using an injection of running buffer for 600 seconds. Following the dissociation step, regeneration of the anti-human IgG Fc surface was performed using 1 injection of glycine•HCl pH 2.0 (Cytiva) at 30 µL/min for 40 seconds. The flow rate for association and dissociation was 30 µL/min.

The kinetics traces were reference subtracted using the responses of the reference channel in each cycle and blank subtracted using a zero-concentration cycle. Then the kinetics constants *k _a_*, *k _d_* and *K _D_* values were determined using Biacore T200 evaluation software and *TitrationAnalysis* tool.

### Octet Red384 data collection

BLI measurements were made using ForteBio biosensors (Fortebio - Sartorius). Both the Data Acquisition 12.0 and Data Analysis 12.0 software packages used were United States Food and Drug Administration’s (FDA) Title 21 Code of Federal Regulations (CFR) Part 11 (FDA Title 21 CFR Part 11) compliant versions. All data collection were performed at 25°C using settings of Standard Kinetics Acquisition rate (5.0 Hz, averaging by 20) at a sample plate shake speed of 1000 rpm. CH31 was loaded onto Anti-human IgG Fc Capture (AHC, Part# 18-5060) sensors with a Δλ = 0.5 nm loading threshold. The AHC sensors loaded with CH31 were then dipped into 1x kinetics buffer (ForteBio, Part# 18-1105) for 60 seconds to obtain baseline and then dipped into wells containing AE.A244 gp120 at different concentrations in 1X kinetics buffer to monitor antibody association. The dissociation step was monitored for 900 seconds by dipping Ab-bound sensors back into the wells used for baseline measurements to facilitate inter-step correction.

Antigen specific binding responses were obtained by double referencing; subtracting responses of blank AHC sensors tested in parallel and 1X kinetics buffer. The specific binding responses were fitted using ForteBio Data Analysis 12.0 software and *TitrationAnalysis* tool.

### Titration data fitting

All sensorgrams were fitted using 1:1 binding model. For fitting of Biacore T200 and Octet Red384 data using *TitrationAnalysis* tool, data was thinned to one data point per second before fitting. During the fitting for data from Carterra LSA and Octet Red 384, signal shift at the beginning of dissociation was not fitted for when using either the commercial software (Carterra Kinetics software and Data Analysis 12.0) or the *TitrationAnalysis* tool. During the fitting for data from Biacore T200, signal shift at the beginning of dissociation was fitted for when using both the commercial software (Biacore T200 Evaluation Software) and the *TitrationAnalysis* tool.

## Results

### Parameter estimates from *TitrationAnalysis* matched well with outputs of commercial software

In order to assess the quality of results generated by the *TitrationAnalysis* tool, we collected binding titration data of HIV-1 envelope glycoproteins AE.A244 gp120 binding to HIV-1 neutralizing monoclonal antibody (mAb) CH31. The binding titration data were collected on Carterra LSA, Biacore T200 and Octet Red384. Non-regenerative cycles were used when collecting data on Carterra LSA.

**Figure 5.**
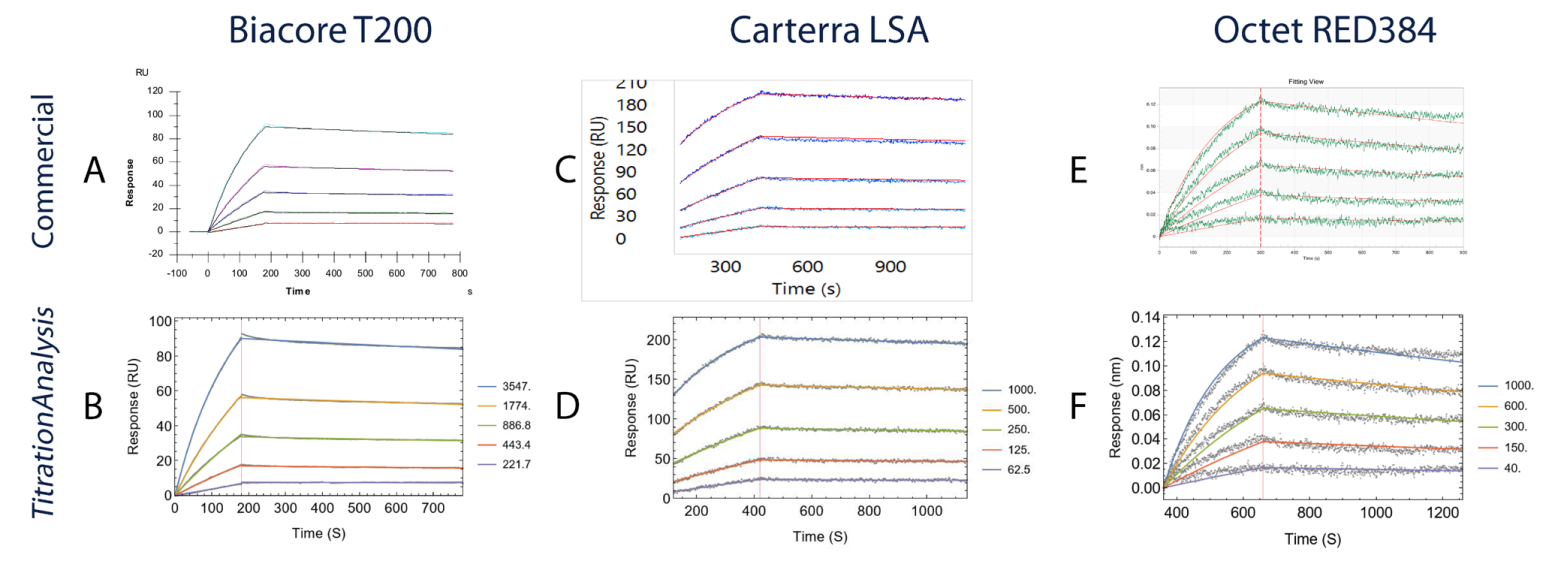
Comparison of fitted sensorgrams obtained using commercial software with the *TitrationAnalysis* tool fitted sensorgrams. Each panel from **A** to **F** shows the binding of AE.A244 gp120 to mAb CH31. Data collected on Biacore T200 are compared in **A** (Biacore T200 evaluation software) and **B** ( *TitrationAnalysis*); data collected on high-throughput SPR _i_ platform Carterra LSA are compared in **C** (Carterra Kinetics software) and **D** ( *TitrationAnalysis*); data collected on Octet RED384 are compared in **E** (ForteBio Data Analysis software) and **F** ( *TitrationAnalysis*).

Figure 5 and Table 2 show the comparison of fitted sensorgram and parameter estimates. To shorten the fitting time, the data collected on Biacore T200 and Octet RED384 were thinned to 1 Hz (one data point per second) when fitting using the *TitrationAnalysis* tool. The data collection frequency of Carterra LSA was ~ 0.5 Hz (about 1 data point per 2 seconds).

**Table 2.** Estimated kinetics by *TitrationAnalysis* closely match those estimates from commercial software. Comparisons of kinetics parameters and their associated standard errors between commercial software analysis and *TitrationAnalysis* tool analysis are shown for all 3 platforms for the binding of AE.A244 gp120 to CH31. “Number of data points” columns indicate the total number of data points used during parameter estimation for each fit.

		Biacore T200 (reference and blank subtracted)				Carterra LSA (reference and blank subtracted)				Octet RED384 (reference subtracted)			
		Number of points	*k _a_* (M ^-1^ s ^-1^)	*k _d_* (s ^-1^)	*K _D_* (M)	Number of points	*k _a_* (M ^-1^ s ^-1^)	*k _d_* (s ^-1^)	*K _D_* (M)	Number of points	*k _a_* (M ^-1^ s ^-1^)	*k _d_* (s ^-1^)	*K _D_* (M)
**Commercial**	**Estimates**	3.9E+04	1.87E+03	1.22E-04	6.54E-08	2.4E+03	3.51E+03	5.87E-05	1.67E-08	2.3E+04	5.68E+03	2.95E-04	5.19E-08
	(associated error)		4.10E+00	2.50E-07	1.96E-10		6.70E+01	2.70E-06	8.32E-10		7.47E+01	4.58E-06	1.06E-09
***Titration*** ***Analysis***	**Estimates**	3.9E+03	1.81E+03	1.19E-04	6.57E-08	2.4E+03	3.71E+03	5.99E-05	1.61E-08	4.5E+03	5.68E+03	2.94E-04	5.17E-08
	(associated error)		8.73E+00	8.04E-07	5.45E-10		9.07E+01	1.14E-06	4.99E-10		7.50E+01	4.59E-06	1.06E-09

For Octet RED384 platform data, the kinetics estimates and the associated standard errors between the commercial software and the *TitrationAnalysis* tool are essentially indistinguishable. For the other two platforms, the estimates also closely resemble between commercial software and the *TitrationAnalysis* tool.

For Biacore T200 platform data, the kinetics estimates of the *TitrationAnalysis* tool showed less than 4% differences when compared to estimates from the commercial software, with the largest being *k _a_* (3.2%). With the commercial software fitting data obtained at 10 Hz and the *TitrationAnalysis* tool fitting data obtained at 1 Hz, the standard errors from the *TitrationAnalysis* fitting only showed modest increase (2.13 – 3.22 fold) and were negligible compared to the estimates (%CV < 1%).

For Carterra LSA platform data, the kinetics estimates for AE.A244 binding showed less than 6% differences between the two fitting methods. Standard errors of *k _d_* and K _D _values associated with *TitrationAnalysis* tool fit were smaller than those associated with commercial software fit. The standard error of *k _a_* from both fits were comparable.

### Replicate measurements of the same interaction yielded similar kinetics estimates

Next, we assessed whether the *TitrationAnalysis* tool can be used to compare replicate measurements of the same interactions. Multiple replicate sensorgrams of AE.A244 gp120 binding to mAb CH31 were collected on the Carterra LSA platform and compared.

**Figure 6.**
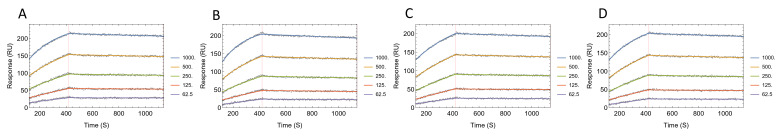
Comparison of fitted sensorgrams for replicate measurements. Panels **A**– **D** show fitted sensorgrams using *TitrationAnalysis* tool for replicates of AE.A244 gp120 binding to mAb CH31. All data were collected on Carterra LSA.

Figure 6 and Table 3 show the comparison of fitted sensorgram and parameter estimates among the replicates. There are small variations among the values, with the largest being 2.11 fold difference among the *k _a_* values of AE.A244 binding to CH31. The level of standard errors is reproducible for the different replicates. Here, the *TitrationAnalysis* high throughput capacity is utilized, and all replicates of a given gp120 species was analyzed in a single run. During this fitting run using the optimized analyte concentration range, the average fitting time for each sensorgram was about 6 seconds.

**Table 3.** Estimated kinetics by *TitrationAnalysis* were similar among replicates. Comparisons of kinetics parameters and their associated standard errors for the replicate measurements of AE.A244 gp120 binding to mAb CH31 using *TitrationAnalysis* tool are shown. SE is abbreviation for standard error. “Fold” indicates the fold change between the largest value and the smallest value among the replicates. All data were collected on Carterra LSA.

Replicate	*k _a_* (M ^-1^ s ^-1^)	SE *k _a_* (M ^-1^ s ^-1^)	fold *k _a_*	*k _d_* (s ^-1^)	SE *k _d_* (s ^-1^)	fold *k _d_*	*K _D_* (M)	SE *K _D_* (M)	fold *K _D_*
1	4.07E+03	1.18E+02	2.11	5.14E-05	1.37E-06	1.51	1.26E-08	4.97E-10	1.68
2	5.50E+03	1.11E+02		7.78E-05	1.38E-06		1.41E-08	3.79E-10	
3	2.61E+03	9.03E+01		5.53E-05	1.13E-06		2.12E-08	8.52E-10	
4	3.71E+03	9.07E+01		5.99E-05	1.14E-06		1.61E-08	4.99E-10	

### The fitting output is not affected by the change in users and machines

To assess whether the fitting output can be reproduced by multiple users implemented on different computers running different Mathematica versions, two users were asked to independently analyze the exact same four sets of Carterra titration data shown in sensorgrams in Figure 5 and Figure 6. The testing was done on two separate computers, with one user using Mathematica 12.2 and another using Mathematica 13.0. The fitting results were compared to the estimates done using Mathematica 12.0 shown in Table 2 and Table 3, and were shown to be highly reproducible ( Table 4). The parameter estimates and the associated errors were indeed identical and independent of the specific computer and software version used.

**Table 4.** The fitting output was reproduced independently by other users. The compilation of fitting results generated by two independent users for AE.A244 binding to CH31 data on all three platforms is shown. SE is abbreviation for standard error.

		Carterra LSA Replicates				Biacore T200	Octet RED384
		Replicate 1	Replicate 2	Replicate 3	Replicate 4		
**User 1**	***k _a_* (M ^-1^ s ^-1^)**	4.07E+03	5.50E+03	2.61E+03	3.71E+03	1.81E+03	5.68E+03
	**SE *k _a_* (M ^-1^ s ^-1^)**	1.18E+02	1.11E+02	9.03E+01	9.07E+01	8.73E+00	7.50E+01
	***k _d_* (s ^-1^)**	5.14E-05	7.78E-05	5.53E-05	5.99E-05	1.19E-04	2.94E-04
	**SE *k _d_* (s ^-1^)**	1.37E-06	1.38E-06	1.13E-06	1.14E-06	8.04E-07	4.59E-06
	***K _D_* (M)**	1.26E-08	1.41E-08	2.12E-08	1.61E-08	6.57E-08	5.17E-08
	**SE *K _D_* (M)**	4.97E-10	3.79E-10	8.52E-10	4.99E-10	5.45E-10	1.06E-09
**User 2**	***k _a_* (M ^-1^ s ^-1^)**	4.07E+03	5.50E+03	2.61E+03	3.71E+03	1.81E+03	5.68E+03
	**SE *k _a_* (M ^-1^ s ^-1^)**	1.18E+02	1.11E+02	9.03E+01	9.07E+01	8.73E+00	7.50E+01
	***k _d_* (s ^-1^)**	5.14E-05	7.78E-05	5.53E-05	5.99E-05	1.19E-04	2.94E-04
	**SE *k _d_* (s ^-1^)**	1.37E-06	1.38E-06	1.13E-06	1.14E-06	8.04E-07	4.59E-06
	***K _D_* (M)**	1.26E-08	1.41E-08	2.12E-08	1.61E-08	6.57E-08	5.17E-08
	**SE *K _D_* (M)**	4.97E-10	3.79E-10	8.52E-10	4.99E-10	5.45E-10	1.06E-09

## Discussion

The overall goal of the *TitrationAnalysis* tool development was to provide flexibility for fitting optimization and reliability of fitting performance, minimize repeated manual interaction with graphic interface and automate the fitting process. Useful features from the three platforms (Biacore T200, Carterra LSA and ForteBio Octet Red384) were incorporated during the development of the *TitrationAnalysis* tool. For example, the fitting for non-regenerative cycles can be applied to data collected on all three platforms. When changing the selection of concentration range or the dissociation window to be used during fitting, there is no need for manual interaction with a graphical interface to exclude titration cycles or adjust fitting window by cropping.

The automation of *TitrationAnalysis* tool primarily aimed at providing a convenient approach to carry out sensorgram fitting in a high-throughput fashion. When assessing the binding kinetics of a diverse panel of ligands binding to the same analyte, it is typically useful to titrate the analyte with a wide concentration range, potentially covering the linear ranges of all ligands. Titration curves corresponding to analyte concentrations that fall under the linear range of dose response typically constitute the best subset of curves in a sensorgram to perform analysis for kinetics estimates, and they contain the least amount of signal artifacts. However, it is laborious to manually determine the linear range of each titration before curve fitting. *TitrationAnalysis* tool provides the ability to automate this process by programmatically finding a range of concentrations that equates to or closely resembles the linear range of each sensorgram. The fitting result using the automatically selected concentration range provides a convenient starting point for fitting optimization.

Currently the development of *TitrationAnalysis* is focused on implementing the 1:1 binding model, which is typically the choice for sensorgram fitting if there is no prior knowledge supporting the need for more complex models. The *TitrationAnalysis* tool fitting equations was adapted to account for the non-zero starting responses at the beginning of the association steps in order to be useful for Carterra LSA data collected non-regeneratively. And the tool does allow user, if needed, to manually select a dissociation window for better fitting of the data to a 1:1 binding model.

Among the output files, the .csv reports of the *TitrationAnalysis* tool can be readily used for statistical calculation and therefore to perform quality control of the data. The PDF reports contains a number of key pieces of information and can be directly used for sensorgram sharing and experiment documentation. This enables laboratories, especially those operating under Good Laboratory Practice (GLP) or Good Clinical Laboratory Practice (GCLP) guidelines, to quickly analyze, document and report results for binding characterization of large panels of biomolecules. We have applied *TitrationAnalysis* to some recent studies, demonstrating its ability for analyzing wide ranges of binding kinetics behavior for large mAb panels, including a panel of SARS-CoV-2 spike protein specific mAbs binding to multiple SARS-CoV-2 spike protein variants ^37^ and a panel of malaria causing *Plasmodium falciparum* circumsporozoite (CSP) protein specific mAbs binding to CSP epitope peptides ^38^.

In the future, the *TitrationAnalysis* tool and its underlying equations can be relatively easily adapted to analyze data from other label free platforms, given that the pre-processed data can be exported from the commercial software. The tool can potentially automate or integrate additional useful sensorgram analysis practices such as more accurate identification of dose response linear range, as well as automatically detecting upward drift in dissociation or biphasic and multiphasic dissociation in order to determine the optimal dissociation fitting window or the appropriateness of using the 1:1 fitting model. We also plan to incorporate steady-state analysis in which the apparent *K _D_* is estimated using the dose response curve. Steady-state analysis requires the estimation of R _eq_ (the response at equilibrium), which has not been reliably established for non-regenerative titrations. Further establishing the methods for R _eq_ estimation can help provide side-by-side comparison of *K _D_* estimated through sensorgram fitting and through steady-state analysis.

Additional binding models beyond 1:1 binding can also be implemented and integrated into the data analysis of multiple platforms. These models use two or more sets of association rate constant and dissociation rate constant to describe a single sensorgram, therefore requiring more rigorous algorithm development. For example, one of our recent endeavors showed that parameter initialization and the length of the dissociation phase can both influence the accuracy of parameter estimation for bivalent analyte model ^39^. Future algorithm development of other non-1:1 binding models and optimization of algorithm performance will benefit the integration of these binding models into the current high-through analysis pipeline.

## Data Availability

Zenodo: Example data for all sensorgrams included in the result section data sets in https://zenodo.org/record/7998652 ^33^. Data are available under the terms of the Creative Commons Attribution 4.0 International license (CC-BY 4.0).

## Software availability

**Source code available from**: https://github.com/DukeCHSI/TitrationAnalysis.

**Archived source code at time of publication:** https://zenodo.org/record/7998652 ^33^

**License:** Creative Commons Attribution 4.0 International license (CC-BY 4.0).
