# Supplementary material for: TitrationAnalysis: a tool for high throughput binding kinetics data analysis for multiple label-free platforms
Source: Gates Open Res. 2024 Jun 28;7:107. Originally published 2023 Jul 4. [Version 2] doi: 10.12688/gatesopenres.14743.2 (PMC10667272; doi:10.12688/gatesopenres.14743.2)
Supplement: Peer review history

# Reviewer report: Reviewer response for version 2

**DOI:** 10.21956/gatesopenres.17408.r38889

**Published:** 2025-01-03

**Authors:** Jason Baardsnes

**Affiliations:** 1 National Research Council Canada, Montréal, Canada

The authors developed third party software intended to process large sets of label-free interaction data and produce output that can be used for reports that conform to GCLP criteria.  At the time of this review, data from Cytiva Biacore T200, Sartorius Octet Red 384, and Carterra LSA are supported by this software.

**Comments:**

 The authors mention other forms of label free interaction technology in the introduction, so many will be awaiting a generic instrument module to import a CSV file of data from some of these other instruments and models not currently supported directly. There are many other technologies not mentioned, including quartz microbalance, surface acoustic wave technology, DNA cantilevers, the list goes on. It could be worth mentioning a timeline for this feature and have an email alert for users waiting for this option.

 In the introduction the authors mention some of the current third-party software that is available but don’t address the shortcomings of the analysis software produced by the instrument vendors. Clearly the older third-party software such as Scrubber weren’t designed to handle large data sets, so most of the potential users for the *TitrationAnalysis* software will be interested in it due to shortcomings of the vendor-supplied analysis software.  This seems like it would be the main driver for developing this software and deserves some comments.  For instance, how will this software improve on the use of BiaEval for T200 in analyzing large sets of data?

 The purpose of the *TitrationAnalysis* software is to process large sets of data, so the authors could show an example of a large screening set of data with a range of KDs and how the output of the data correlates to the output from the instrument analysis software. Additionally, it would be good to show any advantages that *TitrationAnalysis* software has with respect to the workflow with large sets of data compared to using the analysis software from the instrument vendor.

 As a BiaEval software user, the titration subset function of the *TitrationAnalysis* software looks to be a fantastic feature.  It is very arduous to manually select analyte concentrations and process sets of screening data that have a wide range of KDs. Another arduous task is to correct the significant figures in the reporting of the kinetic parameters generated.  The example for BLI data presented on GitHub has about 20 significant figures which is not meaningful.  I would normally report the number of significant figures based of the standard deviation or standard error of a calculated parameter in an analysis.  It would save so much time to be able to control this in the output of a file.

 We currently have both BLI and SPR instruments, so the ability to harmonize their output to a common format for reporting is of interest.  Another advantage of *TitrationAnalysis* not mentioned is the cost of software for multiple users.  Especially for academic labs, the ability to have analysis software on multiple computers from a commercial vendor is cost-prohibitive and could be worth highlighting.

Are the conclusions about the tool and its performance adequately supported by the findings presented in the article?

Yes

Is the rationale for developing the new software tool clearly explained?

Partly

Is the description of the software tool technically sound?

Yes

Are sufficient details of the code, methods and analysis (if applicable) provided to allow replication of the software development and its use by others?

Yes

Is sufficient information provided to allow interpretation of the expected output datasets and any results generated using the tool?

Yes

Reviewer Expertise:

Protein engineering, medium-throughput analysis of biotherapeutics, label free analysis of biomolecules using BLI and SPR technologies.

I confirm that I have read this submission and believe that I have an appropriate level of expertise to confirm that it is of an acceptable scientific standard.

# Reviewer report: Reviewer response for version 2

**DOI:** 10.21956/gatesopenres.17408.r38565

**Published:** 2024-12-31

**Authors:** Janez Ilaš

**Affiliations:** 1 University of Ljubljana, Ljubljana, Slovenia

Authors present Titration Analysis, a high-throughput analysis tool designed to estimate binding kinetics parameters from label-free biophysical techniques like SPR and BLI, using Mathematica's non-linear curve-fitting capabilities. Its importance lies in providing a versatile, automated, and user-friendly solution for biomolecular interaction analysis across multiple platforms, producing reliable results suitable for high-standard laboratory practices.

 The article has undergone significant improvement through previous rounds of review, and I do not have any further corrections to propose

 However, I would like to offer a comment regarding the topic and potential enhancements for the tool in future iterations.

 The authors have validated their software using interactions between two macromolecules, a context highly relevant to industrial applications where surface plasmon resonance (SPR) and biolayer interferometry (BLI) are routinely employed for quality control in antibody production. Automated tools such as this one are particularly valuable in these settings.

 Another important consideration is the potential application of this tool in studying interactions between small molecules and macromolecules, such as in drug discovery. In this field, researchers often encounter the sensitivity limitations of these methods due to the small mass of the analyte (e.g., a potential drug) compared to the large mass of the immobilized protein. Challenges such as a low signal-to-noise ratio or weak analyte binding signals are common. Offering an alternative processing option tailored to address these scenarios would significantly enhance the tool’s utility and broaden its applicability.

 Technical Comment for the Final Version: Figure 5 requires graphical improvements. Specifically, the axes in panels C and E are unreadable and should be reformatted to align with the style of the other panels, ensuring graphical consistency throughout.

Are the conclusions about the tool and its performance adequately supported by the findings presented in the article?

Yes

Is the rationale for developing the new software tool clearly explained?

Yes

Is the description of the software tool technically sound?

Yes

Are sufficient details of the code, methods and analysis (if applicable) provided to allow replication of the software development and its use by others?

Yes

Is sufficient information provided to allow interpretation of the expected output datasets and any results generated using the tool?

Yes

Reviewer Expertise:

Biophysical characterization of interactions between small molecules and macromolecules in drug discovery utilizing techniques such as BLI, SPR, and ITC.

I confirm that I have read this submission and believe that I have an appropriate level of expertise to confirm that it is of an acceptable scientific standard.

# Reviewer report: Reviewer response for version 2

**DOI:** 10.21956/gatesopenres.17408.r39065

**Published:** 2024-12-28

**Authors:** Timothy Bates

**Affiliations:** 1 Oregon Health & Science University, Portland, USA

Li et al. present an updated version of their TitrationAnalysis package for Mathematica which takes data exported from common SPR and BLI platforms to calculate kinetic binding parameters. Open-source analysis tools such as this one are tremendous assets to the research community and TitrationAnalysis take full advantage of the interchangeability of response data from these methods to make it broadly useful across platforms. This also extends the abilities of existing solutions, as current BLI software does not support kinetic titration experiments without regeneration.

 The mathematical models used are correct for this use case, and they are correctly implemented in the code. The use of Mathematica may unfortunately prevent some users from accessing this open-source tool, due to the significant cost of obtaining a license if not provided by an institution/employer.

 I was easily able to reproduce the analysis from the provided example data in Mathematica 14.1, and with some effort I was also able to successfully run their analysis on my own BLI data generated on an Octet Red384 and pre-processed using Data Analysis HT 10.0. The primary challenges were due to apparent differences in the exported data files output by my (slightly older) version of the Octet software, and it required extensive trial and error to manually change the files into a format that was usable by this package. Additional documentation and more descriptive error messages would assist with this process, which would make the code more robust and adaptable to future format changes. Also, more comments in the code itself would substantially help with readability. The text should also make clear that (at least for BLI), the data must already be partially processed (baseline and reference subtraction) before analysis and that this is intended to be done in the Octet software. However, after establishing a suitable method for setting up the input data files, the analysis worked exactly as described and produced a final *K _D_* value within 5% of that produced by the commercial Octet software.

 Overall, this is already a very useful tool for binding kinetics analysis, and it would take only a few quality-of-life changes to make it a great one.

Are the conclusions about the tool and its performance adequately supported by the findings presented in the article?

Yes

Is the rationale for developing the new software tool clearly explained?

Yes

Is the description of the software tool technically sound?

Yes

Are sufficient details of the code, methods and analysis (if applicable) provided to allow replication of the software development and its use by others?

Yes

Is sufficient information provided to allow interpretation of the expected output datasets and any results generated using the tool?

Yes

Reviewer Expertise:

Antibody development/discovery, antibody response to vaccination/infection, biochemical analysis, and host-pathogen interactions.

I confirm that I have read this submission and believe that I have an appropriate level of expertise to confirm that it is of an acceptable scientific standard.

# Reviewer report: Reviewer response for version 1

**DOI:** 10.21956/gatesopenres.16069.r35150

**Published:** 2023-11-23

**Authors:** Ruben Luo

**Affiliations:** 1 Stanford University, Stanford, California, USA

This manuscript introduces a new label-free assay data analysis tool Titration Analysis, which can fit the binding time course data and estimate association and dissociation rate constants for determining apparent dissociation constant values.

 Comments to this review article are listed below.

 (1) In Page 4, first paragraph, please check the correctness of Eq3 as it seemed not consistent with what was described. In addition, was "Rdrift term will be dropped if is Rshift term is included" referring to Eq2? If yes, it seemed that the Rshift term was dropped?

 (2) Although the 1:1 Langmuir fitting model is the most reliable one for label-free assays, please elaborate the reason of not including other binding models.

Are the conclusions about the tool and its performance adequately supported by the findings presented in the article?

Yes

Is the rationale for developing the new software tool clearly explained?

Partly

Is the description of the software tool technically sound?

Yes

Are sufficient details of the code, methods and analysis (if applicable) provided to allow replication of the software development and its use by others?

Yes

Is sufficient information provided to allow interpretation of the expected output datasets and any results generated using the tool?

Yes

Reviewer Expertise:

Label-free assays for clinical diagnostics.

I confirm that I have read this submission and believe that I have an appropriate level of expertise to confirm that it is of an acceptable scientific standard.

# Response

**Published:** 2024-05-27

**Authors:** S. Moses Dennison

**Affiliations:** Surgery, Duke University, Durham, North Carolina, USA

Thanks for the review. Our responses are below.

 • **(1) In Page 4, first paragraph, please check the correctness of Eq3 as it seemed not consistent with what was described. In addition, was "Rdrift term will be dropped if is Rshift term is included" referring to Eq2? If yes, it seemed that the Rshift term was dropped?**

**Response: **Thank you for paying close attention to the parameters in the model. Rshift accounts for the bulk shift at the start of the association due to a mismatch between the analyte buffer and the running buffer used for collecting baseline and dissociation data, potentially contributed by buffer components in the high concentration analyte stock before diluting using the running buffer. This bulk shift will typically disappear when association ends, causing a sudden shift of signal between the end of association and beginning of dissociation. Rdrift, on the other hand, accounts for situation where the disconnect between association and dissociation is caused not by bulk shift, but by other factors such as the loss of non-specifically bound analyte. To avoid over-parameterization, the Rdrift term is omitted when Rshift is included.

 We added the following manuscript texts in the section title “Mathematical model for tool development” to hopefully add further clarification:

 “This bulk shift is typically due to a mismatch between the analyte buffer and the running buffer used for collecting baseline and dissociation data, and will therefore typically disappear when association ends. This causes a signal disconnect both at the beginning and at the end of the association phase.”

 • ( **2) Although the 1:1 Langmuir fitting model is the most reliable one for label-free assays, please elaborate the reason of not including other binding models.**

**Response: **While this manuscript is limited to the automation of 1:1 fitting model for high-throughput analysis, we have started to develop algorithms for rigorous parameter estimations of more advanced binding models. For example, we have described the algorithm development of bivalent analyte binding model in Nguyen et al, 2023. Currently, further development for bivalent analyte fitting is needed to optimize the algorithm performance and enable integration with the current tool.

 We added the following texts in the 6 ^th^ (last) paragraph of the Discussion section to mention our work on this direction:

  “These models use two or more sets of association rate constant and dissociation rate constant to describe a single sensorgram, therefore requiring more rigorous algorithm development. For example, one of our recent endeavors showed that parameter initialization and the length of the dissociation phase can both influence the accuracy of parameter estimation for bivalent analyte model. Future algorithm development of other non-1:1 binding models and optimization of algorithm performance will benefit the integration of these binding models into the current high-through analysis pipeline.”

# Reviewer report: Reviewer response for version 1

**DOI:** 10.21956/gatesopenres.16069.r34620

**Published:** 2023-11-23

**Authors:** Robert Horvath

**Affiliations:** 1 Institute of Technical Physics and Materials Science, Budapest, Hungary

This is a valuable contribution to the community. Often, commercial tools employ codes that are challenging to follow in full detail and do not enable cross-platform analysis, such as creating a clear data architecture to analyze large data sets originating from various measurement platforms using AI. Therefore, any contribution in this direction is useful for making the field more comfortable with AI-based problem-solving. I suggest the authors mention grating-coupled interferometry and focal molography, two novel platforms with advantages over the techniques mentioned in the present work.

Are the conclusions about the tool and its performance adequately supported by the findings presented in the article?

Yes

Is the rationale for developing the new software tool clearly explained?

Yes

Is the description of the software tool technically sound?

Yes

Are sufficient details of the code, methods and analysis (if applicable) provided to allow replication of the software development and its use by others?

Yes

Is sufficient information provided to allow interpretation of the expected output datasets and any results generated using the tool?

Yes

Reviewer Expertise:

NA

I confirm that I have read this submission and believe that I have an appropriate level of expertise to confirm that it is of an acceptable scientific standard.

# Response

**Published:** 2024-05-27

**Authors:** S. Moses Dennison

**Affiliations:** Surgery, Duke University, Durham, North Carolina, USA

Thanks for the review. Our response is below.

 • **I suggest the authors mention grating-coupled interferometry and focal molography, two novel platforms with advantages over the techniques mentioned in the present work.**

**Response: **We appreciate this suggestion of mentioning more recent development of label-free kinetics platforms. We added the following texts as the third paragraph of the Introduction section:

 “Recently, there are also newly emerged label-free techniques that have shown to provide unique advantages. Grating-coupled interferometry (GCI) and focal molography are worthy examples, both of which involve the use of a tantalum pentoxide (Ta2O5) thin-film optical waveguide. GCI uses interference-based waveguide sensors: the reference arm of the interferometer is combined with the measurement arm to eliminate phase noise and fluctuations. GCI exhibited high sensitivity and was shown to be particularly useful for low molecular size analyte under 1000 Da. In focal molography, ligands are precisely assembled in to a specific spatial pattern (molecular hologram) to diffract light coherently, leading to the detection of signal change when bound by a specific target. The noncoherent surroundings do not create coherent diffraction signal, therefore greatly reduce the detection of nonspecific binding. This enables the measurements of molecular interaction directly in biological relevant solutions, such as serum or plasma samples, as well as the detection of protein in living cell cultures.”

# Reviewer report: Reviewer response for version 1

**DOI:** 10.21956/gatesopenres.16069.r34378

**Published:** 2023-09-07

**Authors:** Noah Ditto

**Affiliations:** 1 Carterra Inc., Salt Lake City, UT, USA

Overall the authors provide a clearly described third-party tool for analyzing real-time binding data from commercial biosensors. A simple experimental system is used to prove performance of their fitting process in comparison to analysis software associated with commercial biosensors. 

- To improve reader understanding, it would be helpful to include a brief description of Mathematica in the introduction. 
- Part numbers aren't included for reagents, which may hinder reproducibility aspects of this work. Where possible, would recommend citing part numbers, e.g. the specific goat anti-huFc Millipore antibody. 
- On Table 2, the empty cell on the Number of Points column was slightly confusing; possibly consider merging the two rows under that heading to make it clear that value is applying to both rows. 
- Adding to the caption that the values in Table 3 are derived from the LSA would help in clarifying the data source.
- Its great to see the reproducibility in Table 4 for TitrationAnalysis among users. Would ask that the values be confirmed as they are surprisingly identical. Also suggest the language be more pointed in the caption and/or body to emphasize that they are acknowledged as absolutely identical. 
- In the Discussion, the authors may consider commenting on the feasibility of using TitrationAnalysis for steady state affinity determination since it appears these calculations would not require much more effort to implement. Future considerations could also include the analysis of more kinetically diverse systems, such as those with rapid on- and off-rates. 

Are the conclusions about the tool and its performance adequately supported by the findings presented in the article?

Yes

Is the rationale for developing the new software tool clearly explained?

Yes

Is the description of the software tool technically sound?

Yes

Are sufficient details of the code, methods and analysis (if applicable) provided to allow replication of the software development and its use by others?

Partly

Is sufficient information provided to allow interpretation of the expected output datasets and any results generated using the tool?

Yes

Reviewer Expertise:

Biophysical characterization of biomolecules using techniques such as mass spectrometry, calorimetry, light scattering, BLI, and SPR.

I confirm that I have read this submission and believe that I have an appropriate level of expertise to confirm that it is of an acceptable scientific standard.

# Response

**Published:** 2024-05-27

**Authors:** S. Moses Dennison

**Affiliations:** Surgery, Duke University, Durham, North Carolina, USA

Thanks for the review. Our responses to the questions are below.

 • **To improve reader understanding, it would be helpful to include a brief description of Mathematica in the introduction.**

**Response: **We agree that adding descriptions for Mathematica can help providing contexts for the readers. We have now added the following texts in the 7 ^th^ (last) paragraph of the Introduction section:

  “Mathematica is a software with robust computation abilities and was chosen here as the scripting environment for the TitrationAnalysis tool due to its broad accessibility, particularly to academic researchers.”

 • **Part numbers aren't included for reagents, which may hinder reproducibility aspects of this work. Where possible, would recommend citing part numbers, e.g. the specific goat anti-huFc Millipore antibody.**

**Response: **We agree with this and we have now added the catalog numbers or part numbers for key reagents purchased commercially and for the BLI sensors and SPR chips used for data collection. These changes are included in the sections titled “Carterra LSA data collection”, “Biacore T200 data collection” and “Octet Red384 data collection”.

 • **On Table 2, the empty cell on the Number of Points column was slightly confusing; possibly consider merging the two rows under that heading to make it clear that value is applying to both rows.**

**Response: **We agree that merging the cells will help improve clarify. We have now merged the cells in Table 2 so that the “Number of Points” column for each fit applies to both the parameter estimates and associate errors.

 • **Adding to the caption that the values in Table 3 are derived from the LSA would help in clarifying the data source.**

**Response: **We have now added “All data were collected on Carterra LSA.” in the caption of Table 3.

 • **Its great to see the reproducibility in Table 4 for TitrationAnalysis among users. Would ask that the values be confirmed as they are surprisingly identical. Also suggest the language be more pointed in the caption and/or body to emphasize that they are acknowledged as absolutely identical.**

**Response: ** Thanks for pointing out that the reproducibility was not sufficiently described. Since the same data sets were used by different users, the estimated parameters being identical is not unexpected. The table was indeed used to show that the parameter estimations are not influenced by computers or versions of Mathematica used. In the subsection titled “The fitting output is not affected by the change in users and machines”, we clarified with the following changes:

 “… two users were asked to independently analyze the exact same four sets of Carterra titration data shown in sensorgrams in Figure 5 and Figure 6. The testing was done on two separate computers, with one user using Mathematica 12.2 and another using Mathematica 13.0.”

 We also added the following texts:

 “The parameter estimates and the associated errors were indeed identical and independent of the specific computer and software version used.”

 • **In the Discussion, the authors may consider commenting on the feasibility of using TitrationAnalysis for steady state affinity determination since it appears these calculations would not require much more effort to implement. Future considerations could also include the analysis of more kinetically diverse systems, such as those with rapid on- and off-rates.**

**Response: **Thanks for suggesting the addition of steady state analysis. In order to incorporate steady state analysis, accurate procedure for the estimation of R _eq_ is needed. Currently, the estimation of R _eq_ has not been reliably established for non-regenerative titrations. These capabilities can be incorporated in the upcoming versions.

 We also agree that a more diverse range of kinetics should be tested for the performance of *TitrationAnalysis*. We limited the scope in this article to the binding of AE.A244 to CH31 to fully explore the effective of *TitrationAnalysis*. We actually have demonstrated the usefulness of *TitrationAnalysis* in Li et al, 2024 for analyzing the binding of SARS-CoV-2 spike protein specific antibodies and in Williams et al 2024 for analyzing the binding of circumsporozoite protein specific antibodies that showed diverse range of kinetics.

 Regarding steady state analysis, we have now added the following texts in the 5 ^th^ paragraph of the Discussion section:

 “We also plan to incorporate steady-state analysis in which the apparent *K _D_* is estimated using the dose response curve. Steady-state analysis requires the estimation of R _eq_ (the response at equilibrium), which has not been reliably established for non-regenerative titrations. Further establishing the methods for R _eq_ estimation can help provide side-by-side comparison of *K _D_* estimated through sensorgram fitting and through steady-state analysis.”

 Regarding the analysis of kinetically diverse systems, we have now added the following texts in the 4 ^th^ paragraph of the Discussion section:“We have applied *TitrationAnalysis* to some recent studies, demonstrating its ability for analyzing wide ranges of binding kinetics behavior for large mAb panels, including a panel of SARS-CoV-2 spike protein specific mAbs binding to multiple SARS-CoV-2 spike protein variants and a panel of malaria Plasmodium falciparum circumsporozoite (CSP) protein specific mAbs binding to CSP epitope peptides.”
